# Exploring the limitations of simulator independence via an implementation of a biophysically detailed cerebellar cortex model in NEURON and NEST

**DOI:** 10.1186/1471-2202-14-S1-P93

**Published:** 2013-07-08

**Authors:** Thomas G Close, Ivan Raikov, Shyam Kumar, Erik De Schutter

**Affiliations:** 1 Computational Neuroscience Unit, Okinawa Institute of Science and Technology, Okinawa, 904-0413, Japan; 2 University of Antwerp, Antwerp, 2000, Belgium

## 

The ability to develop models of complex neural networks in a simulator independent manner has been a longstanding goal of the computational neuroscience community [1,2]. One of several important reasons behind this is because the effect of subtle differences in simulator implementations, the timing of spike propagation for example [3], on the qualitative behavior of networks with complex neuronal models is unclear *a priori*. In addition, the relevance of constraints placed on model design by fundamental assumptions in the simulator architecture and features that are not available in all simulators, such as gap junctions and active dendritic compartments, has not been extensively studied. To begin to address such issues, we investigate differences between NEURON [4] and NEST [5] simulations of a biophysically detailed model of the cerebellar cortex.

Following the approach outlined in [6], the cerebellar cortex model is defined using a custom declarative architecture, which is based on NineML [1] and NeuroML 2.0 [7] where possible, and otherwise extended to meet the requirements of the model. Neuronal dynamics are described using a custom extension to the NineML language for conductance-based dynamics, which is compiled directly into simulator-native model formats [8]. Connections between neuronal populations within the model are generated from a combination of morphologically based [9] and soma-to-soma geometric connectivity rules. These rules are integrated into PyNN framework [10], which handles the appropriate simulator-dependent connection routines.

There are a number of factors that make the cerebellar cortex a good test case to study the effect of simulator disparities. The cerebellar cortex is strongly hypothesized to be involved in the fine-tuning of movement [11], and is therefore likely to be sensitive to spike timing. Also, in previous modeling, the behavior of the granular layer sub-network has been shown to be strongly affected by the Golgi-to-Golgi gap junctions [12], making the cerebellar cortex an interesting system in which to study the implications of different implementations of gap junction connections. Therefore, the differences between the NEURON and NEST implementations of the cerebellar cortex model should lend considerable insight into the practical issues that could limit the development of truly simulator independent models.

## References

[B1] Goddard N Hucka M Howell F Cornelis H Shankar K Beeman D Towards NeuroML: model description methods for collaborative modelling in neuroscience Philos Trans R Soc London [Biol] 2001 356.1412 1209 1228 10.1098/rstb.2001.0910 PMC1088511 11545699

[B2] NineML http://software.incf.org/software/NINEML

[B3] Morrison A Straube S Plesser H Diesmann M Exact subthreshold integration with continuous spike times in discrete-time neural network simulations Neural computation 2007 19 1 47 79 10.1162/neco.2007.19.1.47 17134317

[B4] Carnevale NT Hines ML The NEURON Book 2006 Cambridge Univ Pr

[B5] Gewaltig M-O Diesmann M NEST (Neural Simulation Tool) Scholarpedia 2007 2 4 1430 10.4249/scholarpedia.1430

[B6] Close TG Raikov I Negrello M Kumar S De Schutter E Exploring the functional implications of brain architecture and connectivity: a multi-simulator framework for biophysical neuronal models BMC Neurosci 2012 13 Suppl 1 P150 10.1186/1471-2202-13-S1-P150

[B7] NeuroML 2.0 http://www.neuroml.org/neuroml2.php

[B8] Raikov I De Schutter E The Layer-Oriented Approach to Declarative Languages for Biological Modeling PLoS Comput Biol 2012 8 5 10.1371/journal.pcbi.1002521 PMC3355071 22615554

[B9] Negrello M Raikov I De Schutter E Boundary representation of neural architecture and connectivity BMC Neurosci 2011 12 Suppl 1 59 10.1186/1471-2202-12-S1-P5921699685

[B10] Davison AP Brüderle D Eppler J Kremkow J Muller E Pecevski D Perrinet E Yger P PyNN: a common interface for neuronal network simulators Front Neuroinform 2008 2 11 19194529 10.3389/neuro.11.011.2008 PMC2634533

[B11] Braitenberg V Heck D Sultan F The detection and generation of sequences as a key to cerebellar function: experiments and theory The Behavioral and brain sciences 1997 20 2 229 45 10096998

[B12] de Souza FMS De Schutter E Robustness effect of gap junctions between Golgi cells on cerebellar cortex oscillations Neural Systems & Circuits 2011 1 1 7 10.1186/2042-1001-1-7 22330240 PMC3278348

